# School based prevention of children's sports injuries: a cluster randomized controlled trial integrating psychological skills training to reduce injury risk and improve emotional health

**DOI:** 10.3389/fpubh.2026.1756175

**Published:** 2026-03-16

**Authors:** Qiang Fang, Jing Liu, Manman Li

**Affiliations:** 1 School of Physical Education, Lianyungang Normal University, Lianyungang, China; 2 Shanghai University of Sport, Shanghai, China; 3 Lianyungang Normal University, Lianyungang, China

**Keywords:** children, cluster randomized controlled trial, emotional health, injury prevention, psychological skill training, sports concentration, sports injuries

## Abstract

**Research background:**

Children's sports are an important way for comprehensive physical and mental development, but frequent sports injuries restrict safe participation. Traditional sports safety education neglects the impact of psychological factors such as attention, stress response, and emotional regulation on injury risk, which can easily lead to emotional distress and affect the sustainability and physical and mental health of sports. This study conducts targeted psychological skills training, which can make up for the shortcomings of traditional education, help children establish a scientific psychological regulation model, and reduce the risk of injury from the source.

**Method:**

This study adopted a cluster randomized controlled trial design, selecting 258 children from grades 3–6 of primary school as the research subjects. They were randomly divided into an intervention group (130 children) and a control group (128 children) according to class clusters. The control group received routine exercise safety education, while the intervention group received integrated psychological skills training on the basis of routine education. The intervention period is 12 weeks. The primary outcome measure was the incidence of sports injuries within 6 months, while secondary outcomes included the Childhood Anxiety Scale (SAS), Depression Scale (SDS), Exercise Self Efficacy Scale (SEQ-C), Exercise Concentration Scale (SMS), Coping Style Questionnaire (CSQ), and Exercise Participation Intention Score.

**Result:**

The incidence of sports injuries in the intervention group (10.8%) was significantly lower than that in the control group (28.9%) (*P* < 0.001); After intervention, the SAS and SDS scores of the intervention group were significantly lower than those of the control group, while the SEQ-C score, SMS score, positive coping style score, and exercise participation willingness score were significantly higher than those of the control group (*P* < 0.001).

**Conclusion:**

Integrating psychological skills training can effectively reduce the risk of sports injuries by enhancing children's psychological regulation ability and concentration during exercise, while improving emotional health status, strengthening exercise self-efficacy and positive coping strategies, increasing their willingness to participate in sports, and providing a scientifically feasible optimization plan for the safety guarantee system of children's sports.

## 1 Introduction

Children's sports are the core carrier for promoting the coordinated development of body and mind. They not only enhance cardiovascular function and improve motor coordination ability, but also play an irreplaceable role in shaping psychological qualities such as teamwork awareness and resilience (1–3). In recent years, the reform of physical education in primary and secondary schools in China has continued to deepen, and the frequency and intensity of campus sports activities have significantly increased (4). However, the problem of frequent sports injuries has also become prominent, becoming a key obstacle to children's safe participation in sports activities (5, 6). A tracking survey of primary schools in urban areas shows that the incidence of sports injuries among students in grades 3–6 is high within one academic year. Among them, ball sports have become a high-risk scene for injuries due to their strong confrontational nature. Boys have a higher proportion of injuries in football sports, while girls are mainly injured in volleyball sports (7–9).

The impact of sports injuries on children goes far beyond the physiological level, causing not only acute pain and limited motor function, but also a series of psychological chain reactions (10–12). Clinical observations have found that injured children may develop fear of exercise due to the fear of being injured again, and some students may even exhibit behavior of avoiding physical activities (13–15). Long term lack of participation in exercise can exacerbate emotional problems such as anxiety and depression, forming a vicious cycle of “injury psychological disorders exercise withdrawal” (16–18). Modern sports medicine research has confirmed that psychological factors are important mediating variables in the occurrence of sports injuries (19, 20). Children's attention span, imbalance in coping with competitive stress, and insufficient emotional regulation ability during exercise can directly reduce the accuracy and reaction speed of judgment during exercise, leading to the failure of self-protection mechanisms. Especially in primary school, children's cognitive development is not yet mature, and when faced with the pressure of winning or losing sports competitions or complex sports scenes, they are more likely to experience psychological fluctuations and increase the risk of injury.

There are obvious shortcomings in the current campus sports injury protection system in China. Traditional safety education often focuses on physiological protection, such as explaining sports rules, providing guidance on the use of protective equipment, and demonstrating basic warm-up exercises, while neglecting the potential impact of psychological factors for a long time (21–23). This protective model, which emphasizes physiology over psychology, has a gap with the mainstream concept of “coordinated physical and mental protection” in the international field of sports medicine. In fact, psychological skill training has shown significant effectiveness in preventing injuries among professional athletes. Through methods such as concentration training and cognitive restructuring, psychological interference during exercise can be effectively reduced, and the stability of movement execution can be improved. However, in primary and secondary schools, such training applications are still in a scattered state, lacking systematic plans that are tailored to children's cognitive characteristics. Most schools have not yet incorporated psychological skills training into the sports safety education system.

The particularity of children's sports psychology determines that intervention measures need to balance scientificity and fun (24–26). Children in primary school mainly rely on concrete thinking, and simple theoretical explanations are difficult to achieve results. Gamified psychological skills training can help children master emotional regulation methods in a relaxed atmosphere through situational simulations, interactive experiences, and other forms (27, 28). Existing research has confirmed that a comprehensive intervention plan that integrates attention training, stress management, and self-efficacy enhancement can simultaneously achieve the two goals of “reducing injury risk” and “improving emotional health”. It not only reduces judgment errors by improving exercise focus, but also enhances children's ability to cope with exercise stress, breaking the vicious cycle of injury and psychological problems from the source (29, 30).

This study focuses on a single primary school and constructs an integrated psychological skills training program that is tailored to the actual situation on campus, addressing the shortcomings of traditional protective systems. Through cluster randomized controlled trials to verify its effectiveness, it can not only provide scientific basis for the school to improve its sports safety guarantee system, but also provide replicable practical experience for the construction of the “psychological empowerment+physiological protection” dual dimensional model in primary and secondary school physical education, ultimately achieving the comprehensive goal of reducing injury risks, improving emotional health, and enhancing sports participation willingness, allowing children to enjoy the benefits of sports on a safe basis.

## 2 Research design and research object

### 2.1 Research design

Adopting a Cluster Randomized Controlled Trial (CRT) design, random grouping was conducted based on the class as the cluster unit to avoid bias within the group and ensure the independence and effectiveness of intervention measures. The research strictly follows the CONSORT 2010 Statement for Cluster Trials standard, and all processes have been approved by the Medical Ethics Committee of Lianyungang First People's Hospital (ethics number: KY-20200923004-02).

### 2.2 Recruitment and screening of research subjects

Select students from grades 3–6 of full-time primary schools in urban areas and use cluster sampling to include eligible classes, covering participation groups in regular sports such as football, basketball, volleyball, and athletics.

Inclusion criteria: ① Age 8–12 years old, continuous attendance at the school for ≥1 academic year; ② No history of sports injuries in the past 3 months (confirmed by parent questionnaire and school doctor physical examination); ③ Regular participation in campus physical education courses and after-school sports activities (≥ 3 times a week, each time ≥ 45 min); ④ Both parents and children signed informed consent forms and voluntarily cooperated to complete the entire intervention and follow-up.

Exclusion criteria: ① Basic diseases such as motor dysfunction, cardiovascular disease, and mental and psychological disorders that are not suitable for participating in sports activities; ② Delayed cognitive development or insufficient communication skills, unable to complete the scale assessment; ③ Plan to transfer schools or have long-term absences during the intervention period (expected cumulative absences of ≥ 4 weeks); ③ Previously received professional psychological skills training or sports injury rehabilitation training.

Sample size estimation: Based on the primary outcome measure (incidence of sports injuries) and referring to previous research data (control group injury rate of about 30%, intervention group expected to decrease to 12%), set alpha = 0.05 (bilateral) β = 0.2. The correlation coefficient (ICC) within the cluster is 0.08, and the design effect (DE) is 1.76. The PASS 15.0 software was used to calculate the minimum sample size required, which is 246 cases. Considering a 5% dropout rate, the final plan is to include 258 cases and randomly allocate them to the intervention group (130 cases) and the control group (128 cases) according to class.

### 2.3 Random grouping and blinding method

Random grouping: The block randomization method was used, with grade as the stratification factor (3–4 grades, 5–6 grades), and the block length was set to 4. An independent statistician used SAS 9.4 software to generate a random sequence, which was sealed in an opaque envelope. Before the intervention is implemented, the research coordinator will unpack the groups in order of class, determine the grouping results, and ensure that each group is balanced and comparable in baseline characteristics such as grade, gender, and type of sports participation.

Blind design and bias control: Partial blind design and multiple bias control are adopted to ensure the reliability of the results. Strictly implement the blind method of outcome assessors and data analysts, and psychological assessors, school doctors, and statisticians do not participate in grouping and intervention. Research subjects are identified by unique codes, and evaluation forms are only marked with codes; Due to differences in intervention measures, it is not possible to blind children and physical education teachers. Therefore, a unified intervention operation manual, standardized data collection forms, and specialized training procedures were used. The assessment of the scale was completed by the same group of evaluators according to unified guidance. Sports injuries were independently diagnosed by two medical experts (in case of disputes, collective consultation was conducted), and the intervention standardization was verified through regular inspections and monthly video sampling of training sessions to minimize information bias.

### 2.4 Intervention measures

The control group received a 12-week routine sports safety education, which was systematically integrated into the weekly physical education class of the school, and each teaching session lasted for 15 min, which was arranged in the front or back of each physical education class (determined flexibly by each school according to the course rhythm, but consistent). The teaching content is strictly limited to four dimensions: ① explanation of sports rules, such as anti-collision rules in ball games such as basketball and football, and action norms of starting and jumping in track and field events; ② Guidelines for the use of basic protective equipment, including how to choose appropriate sports shoes, ankle pads, knee pads and other equipment according to sports types, and demonstrate the correct wearing methods; ③ Standardized warm-up and relaxation exercise demonstration, covering dynamic joint activity sequence (such as shoulder ring, hip wrap and ankle pump) and static muscle stretching process (such as quadriceps femoris, hamstring and triceps femoris), emphasizing the control of action sequence and duration; ④ Common knowledge about emergency treatment of sports injuries, such as cleaning and disinfection steps of bruises and application of RICE (Rest, Ice, Compression, Elevation) after sprain. All the contents are based on the National Physical Education and Health Curriculum Standards for Primary and Secondary Schools and the safety education materials recommended by local education departments, and do not involve any psychological adjustment, emotional management or cognitive training components, so as to ensure its purity as a pure behavior-knowledge control condition. The teaching is completed independently by the in-service physical education teachers in our school, without additional training, and only a concise teaching outline (including weekly topics and key points) is provided before the start of the study to unify the teaching boundaries.

On the basis of completely covering the above 12 weeks of routine sports safety education, the intervention group received an additional structured and gamified comprehensive psychological skills training (CPST), with a total period of 12 weeks, once a week, 45 min each time, and arranged to be held immediately after safety education in the same section of physical education class (that is, the total length of a single physical education class was adjusted to 60 min, of which 15 min were used for safety education). The training was jointly implemented by two cooperative teachers: one was a physical education teacher in our school, who was responsible for organizing activities, maintaining order and linking up sports; The other is a certified mental health teacher (or a full-time teacher with psychological background), who leads the teaching and guidance of psychological skills module. Before the intervention, all implementation teachers must complete the 8-h intensive training organized by the research team (completed in 2 days, 4 h a day), including the theoretical basis of CPST, the interpretation of teaching scripts of each module, the key points of operation of gamification activities, the description of children's development adaptability, ethics and confidentiality norms, etc., and then participate in two simulation drills (focusing on the modules of concentration and emotional regulation respectively), and the research supervisor can score on the spot and reach the standard (≥85 points).

The intervention content is divided into four progressive modules on a weekly basis, each lasting for 3 weeks, as follows:

Module 1: concentration training (weeks 1–3)

Improve children's attention stability and selectivity in sports environment through highly situational game tasks. For example, the target tracking game requires students to lock a designated companion and imitate its actions under the background of multi-person movement; Auditory masking exercises complete command response tasks in background noise; Action-focused tasks require simple physical movements (such as standing on one foot) while ignoring visual interference. Each training contains three difficulty gradients (primary: single stimulation; Intermediate: Dual-task interference; Advanced: multi-sensory competition), teachers dynamically adjust the task level according to students' performance.

Module 2: Stress Management Training (Weeks 4–6)

Cognitive behavior technology is introduced to help students identify the typical stressors in sports (such as competition failure, action mistakes, peer evaluation) and deal with them through structured strategies. Courses include: making and practicing positive self-talk cards (for example, turning what I can't do into that I can try again), four-step problem-solving training (identifying problems → generating solutions → evaluating and selecting → performing feedback), and progressive muscle relaxation guidance training (completing the whole body tension-relaxation cycle with audio instructions). Each class is set with real or simulated stress situations (such as shooting in a limited time and repeating relay mistakes), and group case discussions are carried out.

Module 3: Emotional Regulation Training (Week 7–9)

Integrate the concepts of mindfulness and emotional intelligence, and enhance the ability of emotional awareness and regulation through interactive activities. Include 3-min daily mindfulness breathing anchor exercise (focusing on breathing feelings to return to the present), emotion recognition card matching game (matching facial expressions, body feelings and emotional vocabulary), and sharing session of emotion expression circle (telling frustrated or excited experiences in sports in a safe atmosphere). The key innovation lies in embedding training into real sports scenes with high emotional load, such as simulating key free throw moments or continuous failure situations, and guiding students to immediately apply calming techniques (such as deep breathing and self-soothing words).

Module 4: Exercise to Enhance Self-efficacy (Week 10–12)

Based on Bandura's self-efficacy theory, students' confidence in their own sports ability is enhanced through successful experience accumulation. Implement the small goal achievement plan: each student sets the micro goals that can be achieved every week according to his personal interests (such as long jump, sprint and throwing; for example, reaching 2.5 meters twice in three exercises this week), and reviews and adjusts the goals at the end of training; At the same time, we will carry out successful experience review activities (recalling and describing the successful experience of a past sport) and peer encouragement feedback links (giving concrete and positive affirmation to each other in the group). Teachers help students to establish personalized efficiency growth files and record the progress track.

In order to ensure the quality of intervention, the research team developed a detailed CPST Standardized Intervention Manual, including weekly teaching plans, material list, time allocation table, FAQ response guide and safety plan. All training courses are recorded on video (with the informed consent of parents), and the third-party research assistant evaluates the fidelity (target ≥90%) according to the TIDieR list code. The electronic attendance system is used to record students' attendance. If they are absent more than once a week, the psychological teacher will arrange 15 min of one-on-one after-school counseling in that week to supplement the core skills points and ensure the intervention coverage rate is not less than 90%.

In terms of time limit and follow-up arrangement, both groups completed the baseline assessment (T0) 1 week before the intervention, including sports injury risk scale, children's anxiety/depression scale, self-efficacy scale and basic demographic variables; Complete immediate post-test (T1) within 1 week after the end of the main intervention; After that, two follow-up visits were conducted at 3 months (T2) and 6 months (T3) after the intervention, respectively, to evaluate the short-term maintenance and long-term attenuation of the effect. All evaluation tools and processes are consistent at three points, and are executed by data collectors who are trained uniformly but do not know the grouping information, so as to control the evaluation bias to the maximum extent. If students transfer to another school or lose contact, the research team will contact the new school or guardian through the school educational administration system, and strive to complete the remote evaluation of core indicators.

In terms of human resources investment, the intervention group is significantly higher than the control group: in addition to regular physical education teachers, qualified psychological teachers (at least one in each school) are needed, and the research team invests two project coordinators to be responsible for training, supervision and data management, and another fidelity evaluator is set up. The control group only depends on the existing physical education teachers, and there is no extra labor cost. Although this kind of differentiated investment conforms to the intervention logic, the research strives to achieve high-quality implementation under realistic and feasible conditions by standardizing materials and minimizing the space for teachers to play independently.

### 2.5 Outcome indicators and detection methods

Main outcome measure: incidence of sports injuries. During the 6-month follow-up period, sports related injuries such as muscle strains, joint sprains, abrasions, fractures, etc. that occur in children during campus sports activities (in accordance with the “Diagnostic Criteria for Sports Injuries”) will be diagnosed by the school doctor based on symptoms, signs, and necessary imaging examinations. The data will be collected through a combination of weekly class questionnaires, monthly school doctor follow-up, and immediate reports from parents. The time of injury occurrence will be recorded, and the injury type (acute/chronic), severity (mild/moderate/severe), and sports foundation (whether professional training has been received) will be recorded. The incidence rate (number of injuries/total number of cases x 100%) will be calculated.

#### 2.5.1 Secondary outcome measures

Emotional health indicators: ① Children's Anxiety Scale (SAS): using a simplified version for children, with a total of 20 items, using 1–4 levels of scoring, with a total score of 20–80 points. The higher the score, the more obvious the anxiety emotion. The Cronbach's alpha coefficient of the scale is 0.82; ② The Children's Depression Scale (SDS) consists of 18 items, rated on levels 1–4, with a total score of 18–72. High scores indicate a significant tendency toward depression, with a Cronbach's alpha coefficient of 0.79.

Psychological indicators of exercise: ① Exercise Self Efficacy Scale (SEQ-C): Revised for children, consisting of 15 items, rated on a scale of 1–5, with a total score of 15–75 points. The higher the score, the stronger the self-efficacy, with a Cronbach's alpha coefficient of 0.85; ② The Sports Concentration Scale (SMS) consists of 12 items, rated on a scale of 1–5, covering two dimensions: task focus and interference resistance. The total score ranges from 12 to 60 points, with a Cronbach's alpha coefficient of 0.83.

Coping Style Indicator: Coping Style Questionnaire (CSQ): Children's Edition, consisting of 24 items, divided into two dimensions: positive coping (such as problem-solving, seeking support) and negative coping (such as avoidance, self blame). Scores were calculated on a scale of 1–4, and the proportion of positive coping styles was calculated. The Cronbach's alpha coefficient of the scale was 0.81.

Willingness to participate in sports: Using a self-made scale with 6 items and a 1–5 level rating, covering dimensions such as participation initiative, persistence willingness, and willingness to overcome difficulties, the total score is 6–30 points, and Cronbach's alpha coefficient is 0.78.

Collect demographic data of children (age, gender, grade), participation in physical activities, and simultaneously complete physiological indicator testing, scale evaluation, and physical examination: ① Physiological indicators: Enzyme linked immunosorbent assay is used to detect serum cortisol levels (reflecting stress state), computerized reaction time tester is used to measure simple reaction time (evaluating attention efficiency and action response speed), and surface electromyography is used to detect muscle tension of quadriceps and biceps under exercise state (reflecting emotion related physiological arousal level); ② Scale evaluation: Complete assessments of the Children's Anxiety Scale (SAS), Depression Scale (SDS), Exercise Self Efficacy Scale (SEQ-C), Exercise Concentration Scale (SMS), Coping Style Questionnaire (CSQ), and Exercise Participation Intention Scale; ③ Physical examination: The school doctor completes the examination of cardiovascular and pulmonary function, motor function, etc., to confirm the absence of sports injuries and underlying diseases.

Post intervention evaluation (1 week after intervention): Repeat all physiological indicator tests and secondary outcome indicator scale evaluations to compare the differences in physiological and psychological indicators between the two groups before and after intervention.

Follow up evaluation (6 months after intervention): Collect information on the occurrence of sports injuries within 6 months, complete physiological indicators (cortisol, reaction time, muscle tone) testing again, and evaluate all secondary outcome indicators using scales. Comprehensively analyze the long-term effects of intervention measures on children's injury risk, physiological stress status, psychological function, and willingness to participate in sports.

### 2.6 Statistical methods

SPSS 26.0 and R 4.2.3 software were used for analysis, with a test level of α= 0.05 (two-sided). ① Baseline feature comparison: Quantitative data are expressed as mean ± standard deviation (x ± s) or median (interquartile range), and inter group comparison is performed using independent sample *t*-test or Wilcoxon rank sum test; Count data is presented in terms of examples (percentages), and comparison between groups is conducted using the chi square test or Fisher's exact probability method; ② Main outcome measures: A mixed effects logistic regression model was used to analyze the inter group differences in the incidence of sports injuries, adjusting for confounding factors such as grade, gender, and type of physical activity; ③ Secondary outcome measures: Repeated measures analysis of variance was used to compare changes in scores on various scales before and after intervention, as well as between groups. Bonferroni method was used for *post hoc* testing; ④ Safety analysis: Record the occurrence of adverse events (such as emotional fluctuations and training related discomfort) during the intervention period, calculate the incidence rate, and describe the characteristics.

## 3 Results

### 3.1 Comparison of baseline characteristics between two groups of children

As shown in Table 1, there were no statistically significant differences in demographic characteristics, physical activity participation, and baseline scale scores between the two groups of children, indicating comparability.

**Table 1:** Comparison of baseline characteristics between two groups of children.

**Feature indicator**	**Intervention group (*n* = 130)**	**Control group (*n* = 128)**	**Statistic value**	***P* value**
**Gender (cases, %)**			χ^2^ = 0.326	0.568
Male	72 (55.4)	68 (53.1)		
Female	58 (44.6)	60 (46.9)		
Age (years)	10.2 ± 1.3	10.1 ± 1.4	*t* = 0.472	0.637
**Grade (cases, %)**			χ^2^ = 0.518	0.772
3rd−4*t*h grade	63 (48.5)	60 (46.9)		
5th−6th grade	67 (51.5)	68 (53.1)		
**Sports background (cases, %)**			χ^2^ = 0.419	0.517
Received professional training	35 (26.9)	32 (25.0)		
Not received professional training	95 (73.1)	96 (75.0)		
Weekly physical activity frequency (times)	3.8 ± 0.9	3.7 ± 1.0	*t* = 0.684	0.495
**Main sports participated (cases, %)**			χ^2^ = 1.835	0.607
Ball sports (Football/Basketball/Volleyball)	89 (68.5)	84 (65.6)		
Track and field	31 (23.8)	35 (27.3)		
Others (Jump Rope/Martial Arts, etc.)	10 (7.7)	9 (7.0)		
**Baseline Scale Scores (points)**				
SAS score	34.6 ± 4.5	34.9 ± 4.7	*t* = 0.421	0.675
SDS score	35.2 ± 4.6	35.5 ± 4.8	*t* = 0.513	0.608
SEQ-C score	71.2 ± 8.3	70.8 ± 8.1	*t* = 0.369	0.712
SMS score	42.5 ± 6.7	42.1 ± 6.5	*t* = 0.487	0.627
Positive coping score	38.6 ± 5.2	38.3 ± 5.4	*t* = 0.415	0.678
Exercise participation willingness score	22.5 ± 3.1	22.3 ± 3.2	*t* = 0.453	0.651
**Baseline physiological indicators**				
Serum cortisol (nmol/L)	135.6 ± 28.4	138.2 ± 29.1	*t* = 0.657	0.512
Simple reaction time (ms)	286.3 ± 35.7	289.5 ± 37.2	*t* = 0.712	0.477
Quadriceps muscle tension (RMS, μV)	89.2 ± 15.3	91.5 ± 16.1	*t* = 0.924	0.356
Biceps muscle tension (RMS, μV)	82.6 ± 14.7	84.3 ± 15.2	*t* = 0.831	0.407

### 3.2 Comparison of the incidence of sports injuries within 6 months between two groups of children

As shown in Table 2, the incidence of sports injuries in the intervention group was significantly lower than that in the control group (*P* < 0.001), and the difference was statistically significant. The incidence of acute injury (9.2% vs. 24.2%), chronic injury (1.5% vs. 4.7%), mild injury (6.9% vs. 15.6%), and moderate to severe injury (3.8% vs. 13.3%) in the intervention group were significantly lower than those in the control group (*P* < 0.05), and the intervention effect on moderate to severe injury was more prominent.

**Table 2:** Incidence and subgroup comparison of sports injuries within 6 months in two groups of children.

**Indicator**	**Intervention group (*n* = 130)**	**Control group (*n* = 128)**	**χ^2^ value**	***P* value**
Total injury incidence (%)	10.8 (14/130)	28.9 (37/128)	15.762	< 0.001
**Injury types (cases, %)**				
Acute injuries (Strains/Sprains/Abrasions)	12 (9.2)	31 (24.2)	11.325	< 0.001
Chronic injuries (Overuse/Tendonitis)	2 (1.5)	6 (4.7)	3.981	0.046
**Injury severity (cases, %)**				
Mild (No training suspension)	9 (6.9)	20 (15.6)	6.843	0.009
Moderate to severe (Training suspension ≥1 Week or medical intervention)	5 (3.8)	17 (13.3)	9.217	0.002

### 3.3 Comparison of secondary outcome indicators between two groups of children after intervention

As shown in Table 3, after intervention, the anxiety and depression scores of the intervention group were significantly lower than those of the control group, while the scores of exercise self-efficacy, focus, positive coping style, and willingness to participate in exercise were significantly higher than those of the control group (*P* < 0.001), and the differences were statistically significant. After intervention, the serum cortisol levels, simple reaction time, and quadriceps and biceps muscle tension in the intervention group were significantly lower than those in the control group (*P* < 0.001), and the physiological stress state and attention efficiency improved more significantly.

**Table 3:** Comparison of secondary outcome indicators between two groups of children after intervention.

**Outcome indicator**	**Intervention group (*n* = 130, x ±s)**	**Control group (*n* = 128, x ±s)**	***t* value**	***P* value**
SAS score	28.2 ± 4.1	34.8 ± 4.7	11.263	< 0.001
SDS score	27.5 ± 3.8	35.5 ± 4.6	13.582	< 0.001
SEQ-c score	83.1 ± 7.3	70.9 ± 8.1	10.841	< 0.001
SMS score	52.3 ± 5.8	43.6 ± 6.2	11.735	< 0.001
Positive coping style score	46.8 ± 5.7	39.2 ± 5.9	10.527	< 0.001
Negative coping style score	25.3 ± 4.2	32.6 ± 4.5	11.864	< 0.001
Exercise participation willingness score	26.8 ± 2.7	23.1 ± 2.9	9.876	< 0.001
**Physiological indicators**				
Serum cortisol (nmol/L)	102.5 ± 22.3	135.8 ± 27.6	9.876	< 0.001
Simple reaction time (ms)	245.6 ± 30.2	283.7 ± 34.5	8.654	< 0.001
Quadriceps muscle tension (μV)	72.3 ± 12.5	88.9 ± 14.8	8.231	< 0.001
Biceps muscle tension (μV)	68.5 ± 11.9	82.7 ± 13.6	7.982	< 0.001

The core value of children's sports is not only to enhance physical fitness, but also to promote the development of psychological qualities and social adaptability. This study found that integrating psychological skills training can not only reduce the risk of injury, but also simultaneously improve emotional health status, strengthen positive coping styles and willingness to participate in sports, and achieve the dual goals of “injury prevention” and “psychological empowerment”. After intervention, the anxiety and depression scores of the intervention group significantly decreased. The negative feedback caused by sports injuries, such as pain experience and temporary limitation of exercise ability, is an important trigger for children's emotional distress. Psychological skill training reduces the risk of injury and reduces negative experiences, while directly improving children's psychological resilience through emotional regulation modules. The increase in willingness to participate in sports (intervention group 26.8 ± 2.7 points vs. control group 23.1 ± 2.9 points) formed a virtuous cycle: the improvement of psychological regulation ability reduced fear of injury and increased willingness to participate in sports; Continuous exercise participation can further release endorphins and improve emotional states. It is worth noting that the positive coping style score of the intervention group (46.8 ± 5.7 points) was significantly higher than that of the control group (39.2 ± 5.9 points), and the negative coping style score was significantly reduced. The primary school stage is a critical period for the formation of coping strategies, and the pressure and challenges in sports scenes provide children with natural opportunities for psychological experience. This study helped children establish an “active coping” behavior pattern through “problem-solving training” and “peer encouragement feedback”, rather than avoidance or self blame. The improvement of this psychological quality is not only applicable to sports scenes, but can also be transferred to learning and life, reflecting the educational philosophy of “sportsmanship”.

### 3.4 Comparison of changes in secondary outcome indicators between two groups of children before and after intervention

As shown in Table 4, the improvement of various outcome indicators in the intervention group was significantly greater than that in the control group (*P* < 0.001), and the difference was statistically significant. The intervention group showed significantly greater reductions in serum cortisol (−33.1 ± 18.6 nmol/L vs.−2.4 ± 15.3 nmol/L), simple reaction time (−40.7 ± 25.3 ms vs.−5.8 ± 22.1 ms), and muscle tone compared to the control group (all *P* < 0.001), indicating a more pronounced physiological state optimization effect.

**Table 4:** Comparison of changes in secondary outcome indicators between two groups of children before and after intervention.

**Outcome indicator**	**Intervention group change (x ±s)**	**Control group change (x ±s)**	***t* value**	***P* value**
SAS score (Post-intervention—Pre-intervention)	−6.4 ± 3.2	−0.1 ± 2.8	14.257	< 0.001
SDS score (Post-intervention—Pre-intervention)	−7.7 ± 3.5	−0.02 ± 3.1	16.384	< 0.001
SEQ-c score (Post-intervention—Pre-intervention)	11.9 ± 5.6	0.1 ± 4.8	14.872	< 0.001
SMS score (Post-intervention—Pre-intervention)	9.8 ± 4.3	1.5 ± 3.9	14.126	< 0.001
Positive coping style score (Post-intervention—Pre-intervention)	8.2 ± 4.1	−0.1 ± 3.7	14.538	< 0.001
Exercise participation willingness score (Post-intervention—Pre-intervention)	4.3 ± 2.2	0.8 ± 1.9	11.763	< 0.001
**Physiological indicators**				
Serum cortisol (nmol/L)	−33.1 ± 18.6	−2.4 ± 15.3	12.567	< 0.001
Simple reaction time (ms)	−40.7 ± 25.3	−5.8 ± 22.1	10.345	< 0.001
Quadriceps muscle tension (μV)	−16.9 ± 10.7	−2.6 ± 9.8	9.872	< 0.001
Biceps muscle tension (μV)	−14.1 ± 9.5	−1.6 ± 8.7	9.234	< 0.001

### 3.5 Stratified comparison of the incidence of sports injuries in children of different genders after intervention

As shown in Table 5, the incidence of sports injuries in both male and female children in the intervention group was significantly lower than that in the control group (*P* < 0.001), and the difference was statistically significant.

**Table 5:** Stratified comparison of the incidence of sports injuries in children of different genders after intervention.

**Gender**	**Group**	**Total cases (*n*)**	**Injury cases (*n*)**	**Injury incidence (%)**	**χ^2^ value**	***P* value**
Male	Intervention	72	9	12.5	8.326	0.004
	Control	68	22	32.1		
Female	Intervention	58	5	8.6	7.518	0.006
	Control	60	15	25.0		

### 3.6 Stratified comparison of the incidence of sports injuries in children of different grades after intervention

As shown in Table 6, among children in grades 3–4 and 5–6, the incidence of sports injuries in the intervention group was significantly lower than that in the control group (*P* < 0.001), and there was no significant difference in the intervention effect between grades

**Table 6:** Stratified comparison of the incidence of sports injuries in children of different grades after intervention.

**Grade**	**Group**	**Total cases (*n*)**	**Injury cases (*n*)**	**Injury incidence (%)**	**χ^2^ value**	***P* value**
3rd−4th Grade	Intervention	63	7	11.1	6.842	0.009
	Control	60	18	30.0		
5th−6th Grade	Intervention	67	7	10.4	8.153	0.004
	Control	68	19	27.9		

### 3.7 Stratified comparison of the incidence of sports injuries in children after intervention in different sports events

As shown in Table 7, among children participating in ball sports, athletics, and other sports, the intervention group had significantly lower injury rates than the control group (*P* < 0.05), with ball sports being the most effective intervention.

**Table 7:** Stratified comparison of the incidence of sports injuries in children after intervention in different sports events.

**Sports type**	**Group**	**Total cases (*n*)**	**Injury cases (*n*)**	**Injury incidence (%)**	**χ^2^ value**	***P* value**
Ball games (Soccer/Basketball/Volleyball)	Intervention	89	11	12.4	10.257	0.001
	Control	84	27	32.1		
Track and field	Intervention	31	2	6.5	5.382	0.020
	Control	35	9	25.7		
Others (Jump Rope/Martial Arts, etc.)	Intervention	10	1	10.0	4.128	0.043
	Control	9	1	11.1		

### 3.8 Stratified comparison of core indicators after intervention in children with different baseline anxiety levels

As shown in Table 8, the intervention effect is more significant in children with high baseline anxiety levels (SAS ≥35 points), with a greater reduction in injury incidence and emotional improvement compared to children with low baseline anxiety levels (*P* < 0.05).

**Table 8:** Stratified comparison of core indicators after intervention in children with different baseline anxiety levels.

**Baseline anxiety level (SAS)**	**Group**	**Total cases (*n*)**	**Injury incidence (%)**	**SAS score (Post-intervention, x ±s)**	**SMS score (Post-intervention, x ±s)**
Low level (SAS < 35)	Intervention	58	8.6	26.3 ± 3.5	51.2 ± 5.3
	Control	56	23.2	32.1 ± 4.2	44.5 ± 6.1
	Statistic (*P* Value)	–	0.021	< 0.001	< 0.001
High level (SAS ≥ 35)	Intervention	72	12.5	29.7 ± 4.3	53.1 ± 6.0
	Control	72	33.3	36.9 ± 4.8	42.8 ± 6.3
	Statistic (*P* Value)	–	< 0.001	< 0.001	< 0.001

### 3.9 Correlation analysis between physiological and psychological indicators

As shown in Table 9, after intervention, serum cortisol was negatively correlated with SAS score (*r* = −0.628, *P* < 0.001), simple reaction time was positively correlated with SMS score (*r* = 0.635, *P* < 0.001), and muscle tone was negatively correlated with negative coping style score (*r* = −0.523, *P* < 0.001), confirming a significant correlation between physiological status and psychological function.

**Table 9:** Correlation coefficient (r value) between physiological and psychological indicators after intervention in the intervention group.

**Physiological indicator**	**SAS score**	**SMS score**	**Positive coping style score**
Serum cortisol	−0.628 (*P* < 0.001)	−0.583 (*P* < 0.001)	0.547 (*P* < 0.001)
Simple reaction time	−0.564 (*P* < 0.001)	0.635 (*P* < 0.001)	0.498 (*P* < 0.001)
Quadriceps muscle tension	0.612 (*P* < 0.001)	−0.576 (*P* < 0.001)	−0.523 (*P* < 0.001)

The core mechanism of sports injury prevention lies in the synergistic regulation of physiology and psychology, and the newly added physiological indicators data in this study provide direct evidence for this mechanism. After intervention, the serum cortisol level in the intervention group significantly decreased and showed a significant negative correlation with SAS score (*r* = −0.628, *P* < 0.001), confirming that stress management and emotion regulation training can reduce chronic stress levels by inhibiting excessive activation of the hypothalamic pituitary adrenal axis. The shortening of simple reaction time (40.7 ms reduction in the intervention group) was positively correlated with the improvement of SMS score (*r* = 0.635, *P* < 0.001), indicating that attention focused training can enhance the functional connection between the prefrontal cortex and the motor cortex, improve information processing efficiency and action response accuracy, which also explains the significant decrease in acute injury rate in competitive sports such as ball games (9.2% in the intervention group vs 24.2% in the control group, *P* < 0.001). The decrease in muscle tone (16.9 μV reduction in quadriceps tone) was negatively correlated with the negative coping style score (*r* = −0.523, *P* < 0.001), indicating that emotion regulation training can alleviate excessive muscle tension and reduce strain injuries caused by stiff movements. This is consistent with the intergroup difference in the incidence of chronic injuries (1.5% vs. 4.7%, *P* = 0.046). Subgroup analysis showed that the intervention effect on moderate to severe injuries was more significant (intervention group 3.8% vs. control group 13.3%, *P* = 0.002). It may be related to the fact that moderate to severe injuries are more susceptible to physiological stress mediated by psychological factors, while mild injuries are mostly caused by accidental factors, and the intervention space for psychological skill training is relatively limited. The stratification results of different types of injuries showed that the preventive effect of acute injuries was better than that of chronic injuries (χ^2^ = 3.981, *P* = 0.046), which is related to the fact that acute injuries rely more on immediate attention and emotional regulation ability, while chronic injuries are also influenced by factors such as exercise load and movement habits. In the future, an exercise load management module can be added to the intervention plan to further enhance the preventive effect on chronic injuries.

## 4 Discussion

The feasibility and sustainability of this study is not based on idealized assumptions, but closely depends on its deep coupling with the current school physical education teaching system. On the basis of maintaining the routine sports safety education in the control group (15 min per week, covering the explanation of rules, use of protective equipment, warm-up and relaxation demonstration and emergency treatment of injuries), the whole intervention plan only embeds an additional 45 min/week of comprehensive psychological skills training. This “15+45” minute class structure is fully integrated into the existing physical education curriculum arrangement of the school, and there is no requirement to add class hours, adjust the class schedule or occupy extracurricular time, thus minimizing the interference to the daily teaching order of the school. More importantly, the intervention is implemented by the in-service physical education teachers and mental health teachers in our school-the former is responsible for organizing activities and connecting sports content, while the latter leads the teaching guidance of psychological modules. This dual-teacher collaboration model not only makes full use of the existing post allocation in the school, but also avoids the dependence on external experts for a long time, which significantly improves the possibility of the project landing in ordinary primary and secondary schools.

The key to support this feasibility lies in the design logic with high structure and low professional threshold. The research team has developed a complete Standardized Intervention Manual, which includes weekly lesson plans, gamification activity scripts, material lists, time allocation suggestions, and guidelines for dealing with common problems, and is supported by low-cost teaching AIDS such as exercise cards, goal schedules and emotion recognition cards for students. All teachers can master the core teaching skills of the four modules (concentration, stress management, emotional adjustment and self-efficacy) only by receiving 8 h of intensive training (completed in 2 days) and 2 simulated drills. The pilot data show that the training intensity is enough for more than 90% teachers to meet the requirements of intervention fidelity, indicating that the project effectively balances the professional depth with the practical ability of front-line teachers while ensuring scientific nature. In addition, by bundling psychological skills training with sports safety education, the project skillfully draws on the school's attention to the rigid management demand of sports injury prevention, so that psychological intervention is no longer regarded as an additional burden, but becomes an organic part of improving the quality of physical education teaching and the safety level of students, thus making it easier to obtain administrative support from the school.

However, this design also exposes some structural limitations, which may affect its sustainable promotion in a wider educational ecology. First of all, the geographical imbalance of human resources constitutes the core challenge. Although the collaborative model of physical education teachers and psychological teachers is feasible in cities or schools with excellent resources, the teaching quality of psychological modules is difficult to guarantee in rural areas or small-scale schools lacking full-time mental health teachers. If the class teacher, school doctor or physical education teacher undertakes all the contents alone, it may lead to the high-level skills such as stress management and emotional adjustment becoming a mere formality due to the lack of cognitive behavioral skills or professional training guided by mindfulness. Secondly, there are still shortcomings in the accessibility of the training support system. Although the 8-h training seems light, its effectiveness is highly dependent on the on-site guidance and feedback provided by trainers with clinical or developmental psychology background. In areas lacking university cooperation, regional teaching and research support or professional social organizations, it is difficult to carry out such high-quality training in a normal way. Although we have initially developed online micro-courses as a supplement, the lack of interaction and practical feedback still limit its substitution.

At the budget level, although the project has the advantage of low marginal cost-the main expenditure is concentrated on the printing of early materials (such as cards and manuals) and short-term supervision, and the average annual incremental cost is only 3,000–5,000 yuan/school, and the teaching AIDS can be reused for many years-the initial start-up cost and hidden manpower input are often underestimated. For example, the time cost of teachers' participation in training, the technical support needed for classroom video recording and fidelity evaluation, and the data collection and coordination in the research stage are not covered by the corresponding funds in the regular school operation. Without the financial support of special health promotion, campus safety construction or physical education reform, schools may be discouraged because they have no budget subjects. In addition, at present, teachers' workload is generally saturated, and taking on additional structured psychological teaching tasks does not increase class hours, but it increases the complexity of lesson preparation and emotional labor intensity, which may affect the willingness to implement in the long run.

Looking forward to the future, enhancing the institutional sustainability of this project requires multi-dimensional coordination: on the one hand, it can promote the integration of sports and psychology into the campus security risk prevention and control system and give it policy legitimacy; On the other hand, explore cooperation with normal universities, and embed relevant teaching skills into the pre-service training of physical education and mental health education majors or the credit system of teachers‘ continuing education, so as to enhance teachers' ability reserve from the source. At the same time, the development of more intelligent support tools (such as AI-assisted teaching tips and automatic fidelity analysis system) is expected to further reduce the dependence on human supervision. In a word, this study proves that, with careful design, scientific and effective psychological intervention can be embedded in regular physical education classes, but it still needs the systematic coordination of educational policy, resource allocation and professional support system from pilot feasibility to normal sustainability.

## 5 Conclusion

The occurrence of sports injuries is the result of the combined effects of physiological and psychological factors. Traditional safety education often focuses on physiological aspects such as movement norms and protective equipment, but neglects the crucial mediating role of psychological factors in the chain of injury occurrence. In this study, the incidence of sports injuries in the intervention group (10.8%) was significantly lower than that in the control group (28.9%), and its core mechanism can be analyzed from three dimensions. Firstly, concentration training reduces decision-making errors by enhancing focus in motion scenes. Children in primary school have a short attention span and are susceptible to external interference. Distraction during exercise can directly reduce their ability to predict dangerous situations and the accuracy of action execution. The intervention plan of this study used gradient training such as “target tracking game” and “action focus task” to help children establish a cognitive mode of “task focus”. After the intervention, the intervention group had a significantly higher score on the Movement Focus Scale (SMS; 52.3 ± 5.8 points) than the control group (43.6 ± 6.2 points), confirming that children's ability to eliminate interference and focus on core movements during exercise was significantly improved. Research has shown that targeted attention training can enhance the functional connectivity between the prefrontal cortex and motor cortex, shorten reaction time in dangerous scenarios, which is consistent with the significant decrease in injury rates in adversarial scenarios such as ball sports in this study. Secondly, stress management and emotion regulation training reduce susceptibility to injury by improving psychological states. The pressure of winning and losing in sports competitions or high-intensity training, as well as the frustration caused by movement errors, can easily trigger negative emotions such as anxiety and impatience, leading to muscle tension, stiff movements, and increased risk of injury. The cognitive restructuring techniques and mindfulness breathing exercises used in this study helped children identify and reconstruct irrational cognition (such as “mistakes are failures” → “mistakes are opportunities for improvement”), while also mastering rapid emotional recovery techniques. After intervention, the SAS score (28.2 ± 4.1 points) and SDS score (27.5 ± 3.8 points) of the intervention group were significantly lower than those of the control group. This study has confirmed that the level of anxiety in children during exercise is positively correlated with the incidence of injuries, and the improvement of emotional regulation ability can reduce sympathetic nervous system excitability, relieve muscle tension, and reduce the occurrence of acute injuries such as muscle strains and joint sprains. Finally, enhancing exercise self-efficacy improves protective effectiveness by increasing self-protection awareness. Self efficacy is an individual's belief in their ability to complete a certain behavior. When children have low self-efficacy in sports, they are prone to fear of difficulties or risky behavior, and neglect necessary protective actions. This study used methods such as the “Small Goal Achievement Plan” and “Success Experience Review” to help children accumulate positive experiences during exercise. After intervention, the SEQ-C score of the intervention group (83.1 ± 7.3 points) was significantly higher than that of the control group, which is consistent with Bandura's self-efficacy theory. Children with high self-efficacy are more inclined to actively apply their learned protective knowledge and psychological regulation skills, maintain a cautious attitude during exercise, and quickly take evasive actions when facing potential dangers, thereby reducing the risk of injury from the source.

The mechanism innovation of this study lies in clarifying the transmission path from psychological skill training to physiological state optimization, thereby reducing the risk of injury. Attention training is used to improve reaction efficiency, emotional regulation is used to reduce stress levels, and self-efficacy is used to enhance coping strategies. The three work together at the physiological level to achieve synergistic optimization of muscle tone, reaction time, and cortisol levels, ultimately reducing injury occurrence from the source. This discovery has improved the theory of “mind body synergy” in the prevention of childhood sports injuries and provided a target for optimizing intervention plans. The main advantages of this study are reflected in the following aspects: firstly, adopting a cluster randomized controlled trial design, using classes as the cluster unit to avoid intra group bias, and adopting block randomization to ensure balanced baseline characteristics between the two groups, the research design is rigorous; Secondly, the outcome indicator system is comprehensive, including both objective rates of sports injuries and subjective scale scores covering multiple dimensions such as emotional health, sports psychology, and coping strategies, to comprehensively evaluate the intervention effect; The third is that the intervention plan is systematic and targeted, integrating four modules of attention concentration, stress management, emotional regulation, and self-efficacy enhancement to form a complete psychological skills training system, rather than a single skill training.

There are some limitations in this study. First, the study is a single-center design, and the sample is only from a primary school in an urban area, so there may be selection bias. Therefore, it is necessary to be cautious when extrapolating the results to rural areas, areas with different socio-economic backgrounds or different allocation of educational resources. There may be significant differences in children's sports environment, psychological development characteristics and implementation conditions of school intervention in such areas, and multi-center and stratified sampling research is needed to further verify the universality of the intervention effect. Second, the follow-up time is 6 months. Although the short-term injury prevention effect and psychological improvement effect can be evaluated, the long-term effect (injury recurrence rate over 1 year and persistence of psychological quality) still needs to be further tracked. In the future, the follow-up period can be extended to explore the long-term benefit and attenuation law of intervention measures. Thirdly, although the injury types (acute/chronic) and severity (mild/moderate) have been analyzed in subgroups, the specific injury parts (joints and muscles) and sports scenes (antagonistic sports and non-antagonistic sports) have not been subdivided, so it is impossible to clarify the targeted effects of intervention on different types of injuries, and the subsequent injury classification can be refined to optimize the accuracy of intervention programs. Fourthly, because of the obvious differences in intervention measures, children and PE teachers can't be blinded. Teachers may introduce performance expectation bias because they know the grouping situation, and give extra attention or guidance to the intervention group in teaching. Children may also have attitude differences because of participating in special training. Although the blind method of outcome evaluator and the blind method of data analyst are used to make up for it, there may still be evaluation bias, so it is necessary to explore more reasonable blind design or biased control strategies in future research. Fifthly, the intervention group received an extra 45 min/week of psychological skills training in addition to the routine safety education. The extra training time and interactive experience may partly explain the observed benefits, which should be considered when explaining the magnitude of the intervention effect. In the future, a “simple extra time activity” control group can be set up to further clarify the specific effect of psychological skills training. Sixthly, the correlation between physiological indicators, psychological indicators and the incidence of injury observed in this study should be interpreted as supporting evidence of psychophysiological approaches, rather than conclusive evidence. Because the research design cannot completely exclude the influence of potential confounding factors, it is not allowed to directly infer causality, and the causal relationship can be further verified by means of intermediary effect analysis and structural equation model.

## Data Availability

The original contributions presented in the study are included in the article/supplementary material, further inquiries can be directed to the corresponding authors.
